# Diversity, Novelty, and Antimicrobial Activity of Endophytic Actinobacteria From Mangrove Plants in Beilun Estuary National Nature Reserve of Guangxi, China

**DOI:** 10.3389/fmicb.2018.00868

**Published:** 2018-05-04

**Authors:** Zhong-ke Jiang, Li Tuo, Da-lin Huang, Ilya A. Osterman, Anton P. Tyurin, Shao-wei Liu, Dmitry A. Lukyanov, Petr V. Sergiev, Olga A. Dontsova, Vladimir A. Korshun, Fei-na Li, Cheng-hang Sun

**Affiliations:** ^1^Institute of Medicinal Biotechnology, Chinese Academy of Medical Sciences & Peking Union Medical College, Beijing, China; ^2^Research Center for Medicine and Biology, Zunyi Medical University, Zunyi, China; ^3^College of Basic Medical Sciences, Guilin Medical University, Guilin, China; ^4^Department of Chemistry, A.N. Belozersky Institute of Physico-Chemical Biology, Lomonosov Moscow State University, Moscow, Russia; ^5^Skolkovo Institute of Science and Technology, Moscow, Russia; ^6^Gause Institute of New Antibiotics, Moscow, Russia; ^7^Shemyakin-Ovchinnikov Institute of Bioorganic Chemistry, Moscow, Russia

**Keywords:** mangrove plants, endophytic actinobacteria, diversity, antimicrobial activity, novel species

## Abstract

Endophytic actinobacteria are one of the important pharmaceutical resources and well known for producing different types of bioactive substances. Nevertheless, detection of the novelty, diversity, and bioactivity on endophytic actinobacteria isolated from mangrove plants are scarce. In this study, five different mangrove plants, *Avicennia marina, Aegiceras corniculatum, Kandelia obovota, Bruguiera gymnorrhiza*, and *Thespesia populnea*, were collected from Beilun Estuary National Nature Reserve in Guangxi Zhuang Autonomous Region, China. A total of 101 endophytic actinobacteria strains were recovered by culture-based approaches. They distributed in 7 orders, 15 families, and 28 genera including *Streptomyces, Curtobacterium, Mycobacterium, Micrococcus, Brevibacterium, Kocuria, Nocardioides, Kineococcus, Kytococcus, Marmoricola, Microbacterium, Micromonospora, Actinoplanes, Agrococcus, Amnibacterium, Brachybacterium, Citricoccus, Dermacoccus, Glutamicibacter, Gordonia, Isoptericola, Janibacter, Leucobacter, Nocardia, Nocardiopsis, Pseudokineococcus, Sanguibacter*, and *Verrucosispora*. Among them, seven strains were potentially new species of genera *Nocardioides, Streptomyces, Amnibacterium, Marmoricola*, and *Mycobacterium*. Above all, strain 8BXZ-J1 has already been characterized as a new species of the genus *Marmoricola*. A total of 63 out of 101 strains were chosen to screen antibacterial activities by paper-disk diffusion method and inhibitors of ribosome and DNA biosynthesis by means of a double fluorescent protein reporter. A total of 31 strains exhibited positive results in at least one antibacterial assay. Notably, strain 8BXZ-J1 and three other potential novel species, 7BMP-1, 5BQP-J3, and 1BXZ-J1, all showed antibacterial bioactivity. In addition, 21 strains showed inhibitory activities against at least one “ESKAPE” resistant pathogens. We also found that *Streptomyces* strains 2BBP-J2 and 1BBP-1 produce bioactive compound with inhibitory activity on protein biosynthesis as result of translation stalling. Meanwhile, *Streptomyces* strain 3BQP-1 produces bioactive compound inducing SOS-response due to DNA damage. In conclusion, this study proved mangrove plants harbored a high diversity of cultivable endophytic actinobacteria, which can be a promising source for discovery of novel species and bioactive compounds.

## Introduction

The increased prevalence of “ESKAPE” pathogens, along with the rapid development of multidrug resistances became the driving force in new antibiotics discovery (Spellberg et al., 2008; Bassetti et al., 2013; Pendleton et al., 2013; Singh et al., 2017). New types of antibacterial drugs are so extremely limited that clinicians are forced to the situation as “Bad Bugs, No Drugs,” which made novel antibiotic discovery become a very important and urgent issue (Talbot et al., 2006; Boucher et al., 2009; Khan and Khan, 2016). It is well known that actinobacteria, especially the genus *Streptomyces*, are major producers of bioactive compounds, it account for nearly 45% of the total bioactive metabolites produced by microorganisms (Bérdy, 2012; Genilloud, 2017). However, after excavation for many decades, the discovery of new species and new antibiotics from common enviroments is becoming increasingly difficult. On the contrary, it is also becoming increasingly evident that un- and under-explored habitats are rich and new sources of actinobacteria for interesting novel bioactive metabolites, including antibiotics (Rateb et al., 2011; Manivasagana et al., 2014; Jiang et al., 2015; Mohammadipanah and Wink, 2015; Axenov-Gibanov et al., 2016; Hassan and Shaikh, 2017). Microbes have to adapt and evolve in metabolite and genetic level to resist the stress from their habitats, thus, have the capability to synthesis of novel chemicals to carry out special biofunctions and bioactivities (Wilson and Brimble, 2009). In fact, a large number of new bioactive compounds produced by actinobacterial strains from special enviroments have been discovered in recent years. (Wilson and Brimble, 2009; Rateb et al., 2011; Xu et al., 2014; Hassan and Shaikh, 2017).

Mangroves locate in the intertidal zone of tropical and subtropical coastlines and possess a unique environment with highly productive ecosystems, it harbors many kinds of microorganisms including actinobacteria (Jensen et al., 1991; Hong et al., 2009; Xu et al., 2014; Azman et al., 2015; Sun et al., 2017). Since 2007, 66 new species and 8 novel genera of actinobacteria have been isolated and identified from mangrove environments (Biswas et al., 2017; Hamada et al., 2017; Jiang et al., 2017; Law et al., 2017; Li F. et al., 2017; Liu et al., 2017; Sun et al., 2017; Qu et al., 2018). Furthermore, at least 84 new compounds including some “hot molecules,” such as salinosporamides, xiamycins and novel indolocarbazoles, were discovered from mangrove actinobacteria. (Kyeremeh et al., 2014; Xu et al., 2014; Ding et al., 2015; Fu et al., 2016; Han et al., 2016; Mangamuri et al., 2016a,b; Chen et al., 2017; Ye et al., 2017).

Endophytic actinobacteria have become a hot spot with increasing actinobacteria prospecting from a range of plant types (Coombs and Franco, 2003; Tian et al., 2007; Qin et al., 2009; Gohain et al., 2015; Passari et al., 2015; Trujillo et al., 2015; Nalini and Prakash, 2017). Moreover, novel endophytic actinobacteria from various tissues of plants have been increasingly reported, and some produced bioactive metabolites with new chemical structures (Qin et al., 2009; Golinska et al., 2015; Matsumoto and Takahashi, 2017; Sun et al., 2017). Until the present, some studies have implemented to isolate endophytic actinobacteria and their secondary metabolites from mangrove plants (Lin et al., 2005; Hong et al., 2009; Wang et al., 2010; Xie et al., 2011; Ding et al., 2012, 2015; Li et al., 2013, 2016; Xu et al., 2014, 2016; Li F. et al., 2017; Li F.N. et al., 2017). According to Xu et al. (2014), 29 of 34 compounds isolated from mangrove endophytic actinobacteria had novel structures, suggesting that mangrove endophytic actinobacteria have the ability to produce new bioactive metabolites. Even though, more than 10 new endophytic actinobacteria species have been characterized from mangrove plants (Jiang et al., 2017; Liu et al., 2017; Sun et al., 2017), studies on mangrove plants endophytic actinobacteria are still rather scarce when compared to actinobacteria prospecting for mangrove soil or sediment. Our results presented in this article revealed that actinobacteria isolated from mangrove plants are important sources for new species and diverse bioactive compounds, and researches on this area should draw much more attention (Yan et al., 2010; Liu et al., 2016, 2017; Tuo et al., 2016a,b; Jiang et al., 2017).

Beilun River is the boundary river between China and Vietnam and the Chinese part is located in Guangxi Zhuang Autonomous Region. The Beilun Estuary National Nature Reserve was estabished to protect mangrove plants in the year of 2000. The reserve contains rich mangrove flora composed of 14 plant species and is mostly unexplored (Liang et al., 2004). To our knowledge, few studies have focused on the endophytic actinobacterial communities of Beilun Estuary mangrove plants. In this study, the diversity and novelty of cultivable actinobacteria from mangrove plants of the Beilun Estuary National Nature Reserve was investigated and their ability to produce antimicrobial activity against “ESKAPE” was evaluated. Meanwhile, a high-throughput screening model based on double fluorescent protein reporter was also implemented to find strains producing secondary metabolites as ribosome and DNA biosynthesis inhibitors.

## Materials and Methods

### Sampling of Mangrove Plants

Mangrove plants were collected from Beilun Estuary National Nature Reserve (21°36’N, 108°12’E) in Guangxi Zhuang Autonomous Region, China, in July, 2015. A total of 19 tissues including leaves, branches, barks, roots, also flowers, and fruits (if present) were collected from five plants: *Avicennia marina, Aegiceras corniculatum, Kandelia obovota, Bruguiera gymnorrhiza*, and *Thespesia populnea*. After flame sterilization, the cut ends of root and branch were sealed with parafilm, and then all samples were transported to the laboratory and processed within 48 h.

### Isolation of Endophytic Actinobacteria

All plant samples were washed thoroughly with tap water for a few minutes to remove organic debris and soil. After air-drying, the samples were processed according to the five-step sterilization method described by Qin et al. (2009), and ground into powder by using micromill, and distributed on plates containing different isolation media. The plates were incubated at 28°C for 2–5 weeks. Colonies that displayed differentiable morphologies were transferred onto ISP2 agar plates and repeatedly isolated and incubated until pure isolates were obtained. The purified cultures were maintained on ISP2 medium slants at 4°C and stored in 20% (v/v) glycerol suspensions at -80°C. A total of 10 media were used for the isolation of mangrove endophytic actinobacteria (Supplementary Table S1), and actidione (40 mg L^-1^), nystatin (40 mg L^-1^), and nalidixic acid (25 mg L^-1^) were added to the media to inhibit the growth of fungi and Gram-negative bacteria.

The efficacy of the sterilization process was confirmed using methods described by Qin et al. (2009). Briefly, the surface-sterilized tissues were washed three times in sterile distilled water, soaked in sterile water for 1 min with continuous stirring, and then, a 0.1 milliliter aliquot of the last washed water was inoculated onto ISP2 agar plates and incubated at 28°C. Meanwhile, the surface-sterilized tissues were imprinted onto ISP2 agar plates and incubated at 28°C. If no microbial growth was observed on the surface of the media, the sterilization was considered as effective.

### Molecular Identification and Phylogenetic Analysis of Isolates

Genomic DNA was extracted from pure isolates as described by Zhou et al. (2010). Universal primers 27F and 1492R (Lane, 1991) were used for amplification of 16S rRNA gene fragments. Cycling conditions were as follows: initial denaturation at 95°C for 5 min, 35 cycles of 94°C for 1 min, 55°C for 1 min, and 72°C for 2 min, and a final extension of 10 min at 72°C. The PCR products were purified and sequenced on the ABI PRISM 3730XL DNA Analyzer from Life Sciences Solutions Group, Thermo Fisher Scientific (Beijing). The genus-level affiliation of the sequences was validated using available 16S rRNA gene sequences from the EzTaxon-e server^1^ (Kim et al., 2012). Sequence alignment and phylogenetic analysis were carried out using MEGA version 5 (Tamura et al., 2011). Phylogenetic trees were constructed by using the neighbor-joining method (Saitou and Nei, 1987) with bootstrap values based on 1000 replications (Felsenstein, 1985) using MEGA5 program. The 16S rRNA gene sequences of the potential novel isolates were deposited in GenBank under the accession numbers: MG563365–563372, and the partial 16S rRNA gene sequences of the remaining isolates were deposited in GenBank with the following accession numbers: MG563311–563364.

### Antibacterial Activity Screening

Based on phylogenetic and phenotypic characteristics analysis, 63 strains were selected for antimicrobial assay. Each strain was transferred to 500 mL Erlenmeyer flasks containing 100 mL of YIM 38 medium (Jiang et al., 2015) and cultivated for 7 days at 28°C with 180 rpm orbital shaking. The 600 mL fermentation broth obtained from each of the isolates was separated from the mycelium by centrifugation at 4500 rpm at 20°C for 20 min. The supernatants were extracted twice with ethyl acetate (1:1, v/v), and the whole organic layer and 50 mL of water layer were concentrated under vacuum and freeze-dried, respectively, to obtain dried samples. The mycelium were soaked in acetone for 12 h and then filtered, the filtration were concentrated under vacuum to obtain dried samples. Finally, each of three kinds of dried samples was dissolved in 3 mL HPLC grade methanol and used in the antibacterial assay by agar disk diffusion method. The methanol sample (60 μL) was dripped on paper disk (diameter, 5 mm). Meanwhile, 60 μL methanol without sample was used as the negative control, and levofloxacin (10 μL, 0.1 mg/mL) was used as the positive control. After being dried in a hood, the paper disks were transferred to agar plates containing pathogenic bacteria and incubated at 37°C for 24 h. The diameters of the inhibition zones were measured by vernier caliper. The indicator bacteria used for antimicrobial assay were: *Enterococcus faecalis* (*E*. *faecalis*) (ATCC 29212, ATCC 51299), *Staphylococcus aureus* (*S*. *aureus*) (ATCC 25923, 2641), *Klebsiella pneumoniae* (*K*. *pneumoniae*) (ATCC 10031, ATCC 700603), *Acinetobacter baumannii* (*A*. *baumannii*) (ATCC19606, 2799), *Pseudomonas aeruginosa* (*P*. *aeruginosa*) (ATCC 27853, 2774), and *Esch*e*richia coli* (*E. coli*) (ATCC 25922, 2800). Isolate 2774 is resistant to carbapenem and quinolone, isolates ATCC 51299, 2641, and 2799 are resistant to vancomycin, methicillin, and carbapenem, and strains 2800 and ATCC 700603 are both ESLB-producing species, respectively.

### Mechanism of Action Determination

Reporter strain JW5503-pDualrep2 was used as previously described (Osterman et al., 2016). Briefly, 1 ml of the extracts from ethyl acetate layer was dried in hood and 100 μl DMSO was added to each sample, and a total of 63 samples were prepared. A total of 2 μl of each sample were applied to agar plate containing a lawn of the reporter strain. After overnight incubation at 37°C, the plate was scanned by ChemiDoc (Bio-Rad): “Cy3-blot” for RFP and “Cy5-blot” for Katushka2S. Erythromycin and levofloxacin were used as positive controls for ribosome and DNA biosynthesis inhibitors, respectively.

## Results

### Isolation and Diversity of Actinobacteria From Mangrove Plants of Beilun Estuary

**FIGURE 1:**
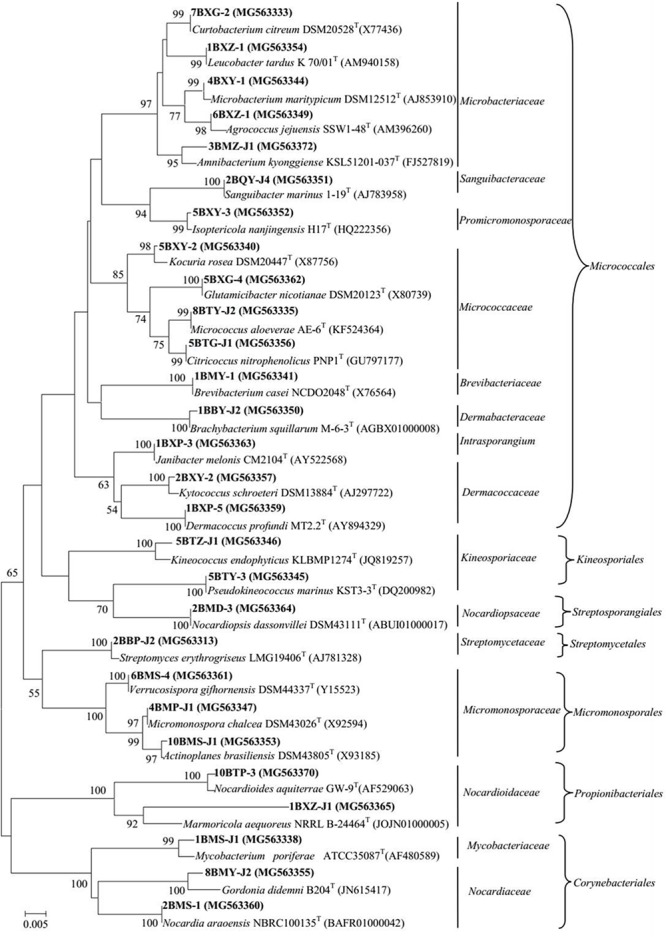
Phylogenetic tree of actinobacterial isolates from mangrove plants of Beilun Estuary that belong to the orders *Micrococcales, Kineosporiales, Streptosporangiales, Streptomycetales, Micromonosporales, Propionibacteriales, Corynebacteriales*, and closely related representative species. Neighbor-joining phylogenetic tree based on 16S rRNA gene sequences of the strains and closely related species of the genera. Numbers at nodes indicate the level of bootstrap support (> 50%) based on 1000 replications. Bar, 5 nt substitutions per 1000 nt.

**FIGURE 2:**
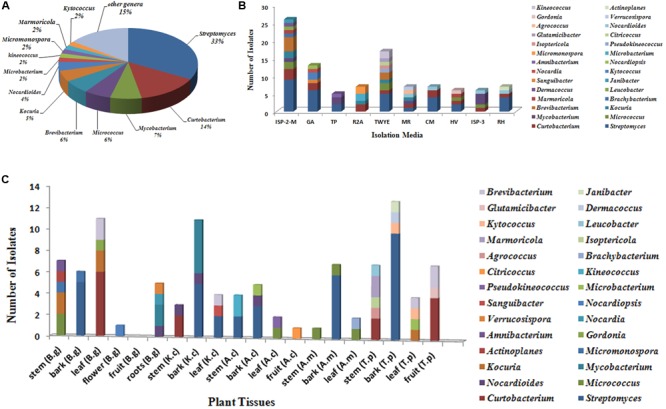
Diversity of culturable actinobacteria from mangrove plants of Beilun Estuary. **(A)** Pie chart representation of the percentage frequency of actinobacterial genera within the total number of isolates. **(B)** Number of actinobacterial isolates recovered from the different culture media used. **(C)** Number of actinobacterial isolates from different tissues of mangrove plants (B.g: *Bruguiera gymnorrhiza*; K.c: *Kandelia candel*; A.c: *Aegiceras corniculatum*; A.m: *Avicennia marina*; T.p: *Thespesia populnea*).

A total of 318 strains of endophytic microbes were isolated from 19 tissues collected from 5 mangrove plants. A total of 158 presumed endophytic actinobacteria were selected on the basis of colonial morphology and were further identified by their 16S rRNA gene sequences. A total of 101 isolates were confirmed as actinobacteria and phylogenetic analysis based on the partial 16S rRNA genes sequences (approximately 700 bp) revealed that the 101 endophytic actinobacterial strains were assigned to 28 genera in 7 orders of 15 families: *Streptomyces, Curtobacterium, Mycobacterium, Micrococcus, Brevibacterium, Kocuria, Nocardioides, Kineococcus, Kytococcus, Marmoricola, Microbacterium, Micromonospora, Actinoplanes, Agrococcus, Amnibacterium, Brachybacterium, Citricoccus, Dermacoccus, Glutamicibacter, Gordonia, Isoptericola, Janibacter, Leucobacter, Nocardia, Nocardiopsis, Pseudokineococcus, Sanguibacter*, and *Verrucosispora* (**Figure 1**). Relative abundance of endophytic actinobacteria at the genus level revealed that *Streptomyces* and *Curtobacterium* were most abundant with 33.0 and 14.0%, respectively (**Figure 2A**). All endophytic actinobacterial strains were isolated from 10 isolation media, which had a major influence on the number of isolates recovered. The ISP2-M medium was the most effective as regards the number and diversity of isolates obtained; the TWYE medium produced the second-highest numbers and diversities. On the contrary, the TP medium yielded the lowest numbers (**Figure 2B**). All tissues produced at least one isolate, which indicated that endophytic actinobacterial isolates can colonize different tissues throughout the plants (**Figure 2C**). Out of 101 isolates, the majority (*n* = 35, 34.65%) were isolated from barks, followed by stem (*n* = 24, 23.76%), leaf (*n* = 22, 21.78%), fruits (*n* = 10, 9.90%), roots (*n* = 9, 8.91%), and flower (*n* = 1, 0.99%).

### Phylogenetic Novelty of Isolated Actinobacteria

**FIGURE 3:**
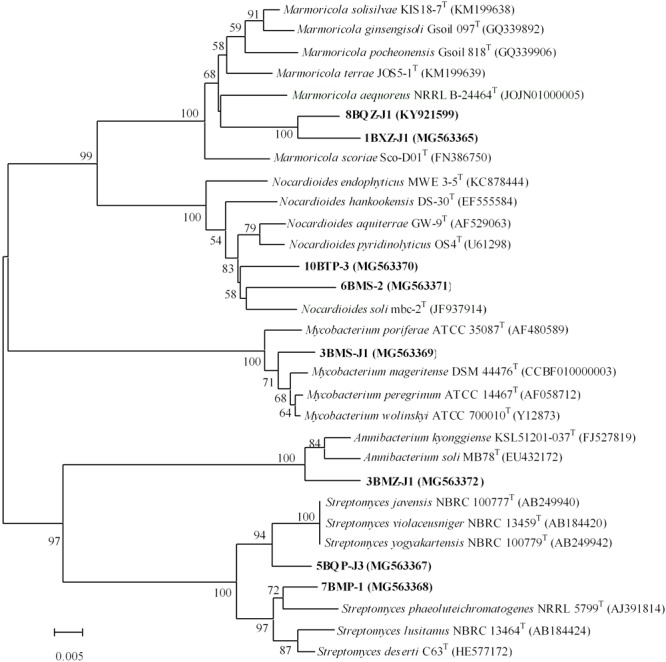
Phylogenetic tree of 7 potential novel strains and 1 new species from mangrove plants of Beilun Estuary and closely related representative species. Neighbor-joining phylogenetic tree based on 16S rRNA gene sequences of the strains and related species of the genera. Numbers at nodes indicate the level of bootstrap support (> 50%) based on 1000 replications. Bar, 5 nt substitutions per 1000 nt.

A total of 7 strains exhibited low sequence similarities (< 98.65%) with validly described species based on a 16S rRNA gene sequence search via the EzTaxon server, suggesting that these strains could represent novel taxons within the phylum *Actinobacteria* (Kim et al., 2014). These putative novel isolates belong to genera *Marmoricola* and *Nocardioides* in family *Nocardioidaceae*, genus *Streptomyces* in family *Streptomycetaceae*, genus *Mycobacterium* in family *Mycobacteriaceae*, and genus *Amnibacterium* in family *Microbacteriaceae*. In addition, strain 8BXZ-J1 has been characterized as a new species of the genus *Marmoricola* (Jiang et al., 2017). The almost complete sequencing (> 1375bp) of the 16S rRNA gene was performed in 7 potential novel strains and the new species. The phylogenetic tree based on 16S rRNA gene sequences generated by using the neighbor-joining method indicated that strain 1BXZ-J1 was clustered within the genus *Marmoricola*, strains 10BTP-3 and 6BMS-J1 were clustered within the genus *Nocardioides*, strains 5BQP-J3 and 7BMP-1 were clustered within the genus *Streptomyces*, strain 3BMZ-J1 was clustered within the genus *Amnibacterium*, and strain 3BMS-J1 was clustered within the genus *Mycobacterium* (**Figure 3**). The 16S rRNA gene sequences of strain 1BXZ-J1, 10BTP-3, 6BMS-J1, 5BQP-J3, 7BMP-1, 3BMZ-J1, and 3BMS-J1 showed highest similarities with *Marmoricola aequoreus* NRRLB-24464^T^ (96.3%), *Nocardioides soli* mbc-2^T^ (97.0%), *Nocardioides aquiterrae* GW-9^T^ (97.3%), *Streptomyces yogyakartensis* NBRC 100779^T^ (97.9%), *Streptomyces phaeoluteichromatogenes* NRRL 5799^T^ (98.2%), *Amnibacterium kyonggiense* KSL51201-037^T^ (98.1%), and *Mycobacterium peregrinum* ATCC 14467^T^ (98.6%), respectively. These isolates will be further characterized in a polyphasic approach to determine their taxonomic positions. The phylogenetic analysis presented implied a considerable cultivable actinobacteria with novelty in mangrove plants from Beilun Estuary.

### Antibacterial Activity of Actinobaterial Isolates

**FIGURE 4:**
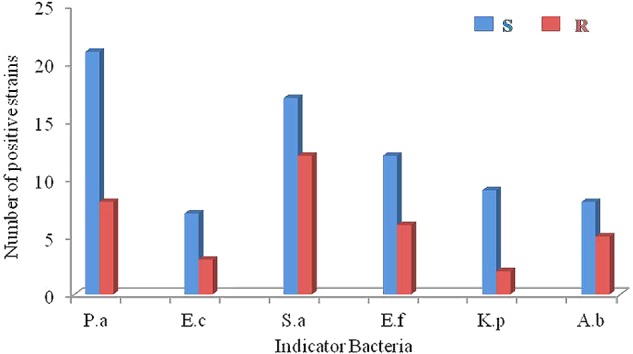
The antimicrobial profiles of the actinobacteria against ESKAPE (P.a: *Pseudomonas aeruginosa*; E.c: *Escherichia coli*; S.a: *Staphyloccocus aureus*; E.f: *Enterococcus faecalis*; K.p: *Klebsiella peneumoniae*; A.b: *Acinetobacter baumannii*). S: sensitive pathogenic strains; R: drug-resistant pathogenic strains.

Antimicrobial activity was evaluated against a set of pathogenic bacteria. Out of 63 isolates, 31 (49.2%) exhibited antagonistic activity against at least one of the tested pathogens (Supplementary Table S2). They were affiliated to 15 different genera, i.e., *Streptomyces* (14), *Curtobacterium* (2), *Micromonospora* (2), *Marmoricola* (2), *Micrococcus* (1), *Nocardioides* (1), *Mycobacterium* (1), *Kocuria* (1), *Microbacterium* (1), *Leucobacter* (1), *Gordonia* (1), *Citricoccus* (1), *Kytococcus* (1), *Nocardia* (1), and *Nocardiopsis* (1). The antimicrobial profile of the actinobacteria against different pathogenic bacteria was shown in **Figure 4**. Regarding the sensitive pathogenic strains tested, activity against *P. aeruginosa* was clearly the most frequent (21 isolates, 33.3%), and activity against *E. coli* was the least frequent (7, 11.1%), while 27.0% (17), 19.0% (12), 14.3 (9), and 12.7% (8) of the isolates were active against *S. aureus, E. faecalis, K. pneumonia*, and *A. baumannii*, respectively. Concerning the resistant pathogenic strains tested, activity against *S. aureus* was the most frequent (12 isolates, 19.0%), and activity against *K. pneumonia* was the least frequent (2, 3.2%), while 12.7% (8), 9.5% (6), 7.9% (5), and 4.8% (3) of the isolates were active against *P. aeruginosa, E. faecalis, A. baumannii*, and *E. coli*, respectively. In all, 21 strains showed inhibitory activities against at least one “ESKAPE” resistant pathogens, suggesting that these strains might represent a valuable source of biologically active compounds with inhibitory activities against “ESKAPE” resistant pathogens.

Notably, three of the seven potential novel strains and new species (8BXZ-J1) showed activity against at least one of the tested pathogenic bacteria. Strain 7BMP-1 exhibited strong antimicrobial activities against *S. aureus* ATCC 25923 (19.1 mm), *S. aureus* 2641 (16.1 mm), *E. faecalis* ATCC 29212 (16.0 mm), *P. aeruginosa* ATCC 27853 (14.0 mm), *E. faecalis* ATCC 33186 (13.8 mm), and *A. baumannii* ATCC 19606 (9.7 mm). Strain 5BQP-J3 showed strong inhibitory activity against *P. aeruginosa* ATCC 27853 (19.1 mm), which is resistant to carbapenem and quinolone. Both strains 1BXZ-J1 and 8BXZ-J1 showed strong inhibitory activity against *P. aeruginosa* ATCC 27853, and the inhibition zones were 19.4 and 17.1 mm, respectively.

**FIGURE 5:**
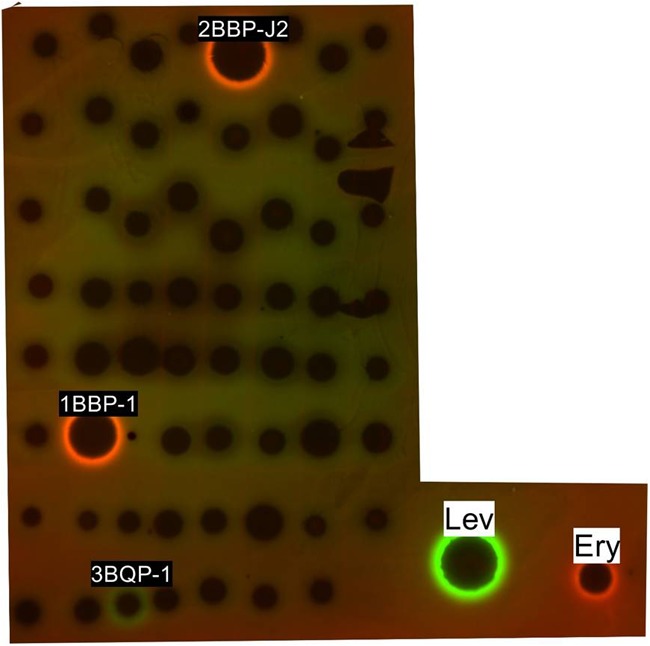
Induction of a two-color dual reporter system sensitive to inhibitors of the ribosome progression or inhibitors of DNA replication, respectively. Spots of erythromycin (ERY), levofloxacin (LEV), and tested compounds were placed on the surface of an agar plate containing *E. coli tolC* cells transformed with the pDualrep2 reporter plasmid. Shown is the fluorescence of the lawn of *E. coli* cells scanned at 553/574 nm (green pseudocolor) for RFP fluorescence and 588/633 nm (red pseudocolor) for Katushka2S fluorescence. Induction of expression of Katushka2S is triggered by translation inhibitors, while RFP is upregulated by induction of DNA damage SOS response.

The 63 ethyl acetate extracts were assayed by a double fluorescent protein reporter (pDualrep2 reporter system) screening model and the results are shown in **Figure 5**. Strains 2BBP-J2 and 1BBP-1 induced Katushka2S expression, which demonstrated strong translation inhibition activity as erythromycin did, meanwhile, strain 3BQP-1-induced RFP expression and SOS-response because of DNA-damage as levofloxacin did.

## Discussion

Mangroves plants harbor a great diversity of culturable actinobacteria, and are proven to be a valuable microorganism source for discovery of new bioactive metabolites (Hong et al., 2009; Xu et al., 2014, 2016; Li et al., 2016; Li F.N. et al., 2017). In this study, a considerable diversity of endophytic actinobacteria was obtained from the mangrove plants collected in Beilun Estuary National Nature Reserve of Guangxi Zhuang Autonomous Region, China. In addition, to our knowledge, this is the first time strains that were rarely recovered in genera *Sanguibacter* and *Citricoccus* have been isolated and cultured from the inner parts of plants. Recently, Li F.N. et al. (2017) reported that 198 endophytic actinobacterial strains isolated from mangrove plants of Macao, China, were distributed in 30 genera affiliated to 8 orders. Li et al. (2016) isolated 146 endophytic actinobacteria belonging to 8 orders (18 genera) from mangrove plants of Dongzhaigang of Hainan Province, China. The 159 isolates of 19 actinobacteria genera belonging to 8 orders obtained from mangrove plants of Zhanjiang in Guangdong Province were reported in 2016 (Xu et al., 2016). Hong et al. (2009) identified 237 actinobacterial isolates belonging to five orders (13 different genera) from soil and plant samples of 8 mangrove sites in China. In our study, we identified 28 genera from 101 isolated actinobacterial strains, the diversity of endophytic actinobacteria is apparently higher than the previous studies. Therefore, it is clear that endophytic actinobacteria in mangrove plants are diverse, and that the variation in the diversity and richness of endophytic actinobacteria recovered from mangrove plants are closely related with the isolation media and different tissues.

Some novel endophytic actinobacteria have been discovered from mangrove plants, suggesting that they have potential as excellent sources of novel species with actinobacterial reagents (Liu et al., 2017; Sun et al., 2017). In this study, isolates from the genus *Streptomyces* were the most abundant, it is consistent with previous studies on actinobacterial communities in mangrove plants (Hong et al., 2009; Xu et al., 2014, 2016; Li et al., 2016; Li F.N. et al., 2017). Two *Streptomyces* strains (5BQP-J3 and 7BMP-1) are probably new species due to their relatively low gene sequence similarities to their closest type strains. Meanwhile, the gene sequence similarity between 5BQP-J3 and 7BMP-1 was 96.5%. In addition, five putative new rare actinobacteria species are proposed: *Nocardioides* sp. 10BTP-3, *Nocardioides* sp. 6BMS-2, *Marmoricola* sp. 1BXZ-J1, *Mycobacterium* sp. 3BMS-J1, and *Amnibacterium* sp. 3BMZ-J1, based on numerical thresholds related to 16S rRNA gene sequences (Kim et al., 2014; Supplementary Table S2). Further phylogenetic analysis of the isolates is shown in **Figure 3**. Pairwise comparison of the 16S rRNA gene sequences from the two *Nocardioides* strains (10BTP-3 and 6BMS-2) showed relatively low similarities (97.4 and 97.0%) to the type strains of *Nocardioides aquiterrae* and *Nocardioides soli*, respectively. The sequence similarity between 10BTP-3 and 6BMS-2 was 97.5%. Furthermore, the phylogenetic analysis suggested that the two isolates were diversely distributed within genus *Nocardioides* and clustered singly in the phylogenetic tree (**Figure 3**), indicating that isolates 10BTP-3 and 6BMS-2 might belong to new species. The *Marmoricola* strains 1BXZ-J1 and 8BXZ-J1 had lineages that were distinct from each other and from other members of the genus; and they also formed distinct subclades in the tree supported by high bootstrap values (**Figure 3**). The 16S rRNA gene sequences of 1BXZ-J1 and 8BXZ-J1 showed 96.3 and 96.9% identities to the nearest neighbors, *Marmoricola aequoreus* and *Marmoricola solisilvae*, respectively. The sequence similarity between 1BXZ-J1 and 8BXZ-J1 was 97.8%. Strain 8BXZ-J1 has been characterized as the first endophytic actinobacteria new species belonged to the genus *Marmoricola* (Jiang et al., 2017). *Mycobacterium* strains have been isolated from mangrove plants (Xu et al., 2016; Li F.N. et al., 2017) and other medicinal plants (Qin et al., 2009). However, novel species of the genus *Mycobacterium* from mangrove ecosystem have not yet been reported. 3BMS-J1 is probably a new *Mycobacterium* species because it presents relatively low 16S rRNA gene sequences similarity and forms a distinct phylogenetic branch when compared to strains from the genus *Mycobacterium* (**Figure 3**). Comparison of the 16S rRNA gene sequences of the isolate and its closest neighbors suggested that 3BMZ-J1 was closely related to the type strains of the genera *Amnibacterium.* To date, this genus comprises only two species that were isolated from Anyang stream and grass soil (Kim and Lee, 2011; Jin et al., 2013). Strain 8BXZ-J1 was isolated with HV medium, the potential novel strains 3BMS-J1 and 3BMZ-J1 were both isolated with TP medium, and the others potential novel strains 1BXZ-J1, 5BQP-J3, 6BMS-2, 7BMP-1, and 10BTP-3 were isolated with ISP-2-M medium, TWYE medium, MR medium, CM medium, and RH medium, respectively. This result demonstrated that it is still worthwhile to use traditional cultivating methods for isolating new actinobacterial species.

Analysis of composition in genera level of 31 positive strains from 63 tested strains indicated that *Streptomyces* strains predominated. In 63 tested strains, there are 15 *Streptomyces* strains, among them, 14 *Streptomyces* strains showed positive result. It is quite reasonable, since *Streptomyces* is the largest antibiotic-producing genus in the microbial world discovered, and it is well documented that mangrove *Streptomyces* are able to produce bioactive metabolites with a wide-range of activities, including antibacterial, antifungal, anti-HIV, and anticancer (Ding et al., 2010; Yuan et al., 2013; Khieu et al., 2015; Tan et al., 2015; Xu et al., 2015; Ser et al., 2017; Wang et al., 2017). *Curtobacterium* as the second most dominant genus in 63 tested strains, showed very low positive rate, only 2 of 11 displayed antibacterial activities against tested pathogens. *Curtobacterium* traditionally is viewed as a plant pathogen, and most studies focused on its role as an economically important plant pathogen (Huang et al., 2009; Buonaurio et al., 2015; Osdaghi et al., 2015). Recently, Undabarrena et al. (2016) reported *Curtobacterium* isolates showing potent antimicrobial bioactivity against more than one tested pathogens, and their antimicrobial activity was dependent on the culturing media.

It is noteworthy to highlight that the two rare actinobacterial strains, *Kytococcus* strain 2BXY-2 and *Leucobacter* strain 1BXZ-1, showed the strong inhibitory activity against *Pseudomonas aeruginosa* as shown in Supplementary Table S2. To our knowledge, antibacterial activities from these genera have been received scarce attention for potential development of novel antimicrobial reagents.

It is extensively accepted that novel microorganisms are a good source for the discovery of new secondary bioactive metabolites, as exemplified by salinosporamide A, a potent cytotoxic activity compound produced by *Salinispora* strain CNB-392 (Feling et al., 2003) and teixobactin, a new cell wall inhibitor with exceptional activity against *Clostridium difficile* and *Bacillus anthracis* produced by *Eleftheria terrae* (Ling et al., 2015). In the study, two potential new *Streptomyces* spieces (5BQP-J3 and 7BMP-1) displayed very strong antibacterial activities (Supplementary Table S2), thus it would be interesting to see if new antibiotics can be identified from these strains. In addition, antibacterial activities of *Marmoricola* isolates (1BXZ-J1 and 8BXZ-J1) against *P*. *aeruginosa* and *S*. *aureus* are highlighted by their novelty. Few have reported the antimicrobial activity potential of *Marmoricola* so far. *Marmoricola* isolates (1BXZ-J1 and 8BXZ-J1) may also be good candidates for production of bioactive compounds. Further experiments will be carried out to deepen our knowledge on the antibacterial activities of these novel isolates.

In the antibacterial assay, pDualrep2 reporter system was implemented to indicate existence of antibiotics in the culture broth, the system can distinguish simultaneously between antibiotics that induce the SOS response, a major general stress response caused by inhibitors of DNA biosynthesis and those that cause ribosome stalling. The existence of ribosome inhibitor such as erythromycin will lead Katushka2S expression, and the existence of inhibitor of DNA biosynthesis such as levofloxacin will lead RFP expression. Both 2BBP-J2 and 1BBP-1 fermentation broth demonstrated ribosome inhibition activities in their ethyl acetate extractions, indicating that Katushka2S expression had been induced. Meanwhile, strain 3BQP-1 demonstrated inhibition activity against DNA biosynthesis in its fermentation broth extract as shown by the induced RFP expression.

The diversity, novelty, and antimicrobial activity of endophytic actinobacteria from mangrove plants of Beilun Estuary were first comprehensively investigated in this study. The 101 selected isolates were assigned to 7 orders, 15 families, and 28 genera. Notably, 31 isolates belonging to 15 different genera show inhibitory activities against at least one “ESKAPE” pathogens, and 7 isolates affiliated with 5 genera are below the 98.65% sequence identity threshold and therefore may be potential candidates of new taxons. In addition, a new species of the genus *Marmoricola* isolated from the mangrove plants has been published. These results indicated that mangrove plants are potentially unique sources of novel actinobacteria with promising potential to produce highly bioactive metabolites.

## Author Contributions

Z-kJ carried out the sampling, the selective isolation, the primary identification of the isolates, the screening of antimicrobial activities, analyzed the data, and prepared the manuscript. LT and D-lH carried out the sampling, the selective isolation, and edited the manuscript. S-wL and F-nL prepared the samples for screening. OD, AT, DL, PS, IO, and VK sorted out the antibiotics’ mechanisms of action of the extracts by means of a double fluorescent protein reporter for high-throughput screening of ribosome and DNA biosynthesis inhibitors and edited the manuscript. C-hS conceived and designed the experiments, performed the sampling, and edited the manuscript.

## Conflict of Interest Statement

The authors declare that the research was conducted in the absence of any commercial or financial relationships that could be construed as a potential conflict of interest.

## Footnotes

1 http://eztaxon-e.ezbiocloud.net
