# Supplementary material for: Diversity, Novelty, and Antimicrobial Activity of Endophytic Actinobacteria From Mangrove Plants in Beilun Estuary National Nature Reserve of Guangxi, China
Source: Front Microbiol. 2018 May 4;9:868. doi: 10.3389/fmicb.2018.00868 (PMC5945994; doi:10.3389/fmicb.2018.00868)
Supplement: Supplementary file 1 [file Table_1.PDF]

Supplementary Material

Diversity, Novelty and Antimicrobial Activity of Endophytic Actinobacteria from Mangrove Plants in Beilun Estuary National Nature Reserve of Guangxi, China

Zhong-ke Jiang[1], Li Tuo[2], Da-lin Huang[3], Osterman I. A[4,5], Anton P. Tyurin[6,7], Shao-wei Liu[1], Lukyanov D. A.[5], Sergiev P.V.[4,5], Dontsova O.A.[4,5], Vladimir A. Korshun[6,7], Fei-na Li[1] and Cheng-hang Sun[1]*

1. Institute of Medicinal Biotechnology, Chinese Academy of Medical Science & Peking Union Medical College, Beijing 100050, China
2. Research Center for Medicine & Biology, Zunyi Medical University, Zunyi 563003, China
3. Guilin Medical University, Guilin 541004, China
4. Lomonosov Moscow State University, Department of Chemistry and A.N. Belozersky Institute of Physico-Chemical Biology, Moscow, 119992, Russia
5. Skolkovo Institute of Science and Technology, Moscow, 143025, Russia
6. Gause Institute of New Antibiotics, Moscow, 119021, Russia
7. Shemyakin-Ovchinnikkov Institute of Bioorganic Chemistry, Moscow, 117997, Russia

*Corresponding author:

Cheng-hang Sun

Postal address: Department of Microbial Chemistry, Institute of Medicinal Biotechnology, Chinese Academy of Medical Sciences, Tian Tan Xi Li No.1, Beijing 100050, China.

Tel: +86 10 63165278;

Fax: +86 10 63017302.

Email address: chenghangsun@hotmail.com (C-H sun) or sunchenghang@imb.pumc.edu.cn

Table S1. Compositions of ten different media used for isolation of mangrove endophytic actinobacteria.

**Medium**	**Composition**
HV	Humic acid (1.00 g); Na_2HPO_4 (0.50 g); KCl (1.70 g); $CaCO_3$ (0.02 g); $FeSO_4$ (0.01 g); 0.00005% each of thiamine HCl, riboflavin, niacin, pyridoxine–HCl, inositol, Ca-pantothenate, p-aminobenzoic acid, and 0.000025% of biotin; Agar (18.00 g); Distilled water (1000.00 ml); pH 6.0
ISP-2-M (YIM 38)	Glucose (2.00 g); Yeast extract (2.00 g); Malt extract (3.00 g); Agar (20.00 g); vitamin mixture (1.00 ml); trace salt solution (1.00 ml); Distilled water 1000.00 ml; pH 6.0
R2A	R2A agar (Difco)
TWYE	Yeast extract (0.25 g); K_2HPO_4 (0.50 g); Agar (18.00 g); Tap water (1000.00 ml)
GA	Soluble starch (20.00 g); KNO_3 (1.00 g); K_2HPO_4 (0.50 g); $MgSO_4.7H_2O$ (0.05 g); ,$FeSO_4 \cdot 7H_2O$ (0.01 g); Agar (20.00 g); Distilled water (1000.0 ml); pH 7.4-7.6
MR	MR agar (Difco)
RH	Raffinose (1.00 g); histidine (1.00 g); Na_2HPO_4 (0.50 g); KCl (1.70 g); $MgSO_4 \cdot 7H_2O$ (0.05 g); $FeSO_4 \cdot 7H_2O$ (0.10 g); $CaCO_3$ (0.02 g); Agar (18.00 g); vitamin mixture (1.00 ml); Distilled water (1000.00 ml); pH 8.0
TP	Trehalose (5.00 g); proline (1.00 g); $(NH_4)_2SO_4$ (1.00 g); NaCl (1.00 g); $CaCl_2$ (2.00 g); K_2HPO_4 (1.00 g); $MgSO_4.7H_2O$ (1.00 g); Agar (20.00 g); vitamin mixture (1.00 ml), Distilled water (1000.00 ml); pH 7.2
CM	Microcrystalline cellulose (10.00 g); Casein (0.30 g); KNO_3 (0.20 g); K_2HPO_4 (0.50 g); $CaCO_3$ (0.02 g); FeSO4 (0.01 g); NaCl (100 g); $MgCl_2$.6H2O (30 g), KCl (20 g); Agar (15 g); Distilled water (1000.0 ml); pH 7.5
ISP-3	Oatmeal (20.00 g); $FeSO_4 \cdot 7H_2O$ (0.001 g); $MnCl_2.4H_2O$ (0.001 g); $ZnSO_4 \cdot 7H_2O$ (0.001 g); Agar (18.00 g); Distilled water (1000.00 ml); pH 7.3

Trace salt solution: $FeSO_4 \cdot 7H_2O$ (0.20 g); $MnCl_2.4H_2O$ (0.01 g); $ZnSO_4 \cdot 7H_2O$ (0.01 g); Distilled water (100.00 ml)

Vitamin mixture: Thiamine (0.10 g); Pyridoxine (0.10 g); Riboflavin (0.10 g); Niacin (0.10 g); Biotin (0.10 g); Distilled water (100.00 ml)

Table S2. Antimicrobial activities and similarity values for 16S rRNA gene sequences of culturable endophytic actinobacteria from mangrove plants.

Isolate (GenBank accession no.)	Genus and species (Similarity, %)	Fractions[a]	Activity[b]											
			P. aeruginosa		*E. coli*		*S. aureus*		*E. faecalis*		*K. peneumoniae*		*A. baumannii*	
			S	R	S	R	S	R	S	R	S	R	S	R
1BMP-J1	*Streptomyces wuyuanensis*	E	–	–	–	–			–	–	–	–	–	–
(MG563311)	CGMCC 4.7042[T] (99.29%)	M	–	–	–	–	-	–	–	–	–	–	–	–
		A	–	–	–	–	–	-	–	–	–	–	–	–
2BBP-J2	*Streptomyces erythrogriseus*	E	–	–	–	–	1.11	0.68	0.67	–	–	–	0.72	–
(MG563313)	LMG 19406[T] (99.86%)	M	–	–	0.69	0.74	0.88	1.26	–	–	0.57	-	–	-
		A	–	–	–	–	–	–	–	–	-	–	–	–
5BQP-J3	*Streptomyces yogyakartensis*	E	–	–	–	–	–	–	–	–	–	–	–	–
(MG563367)	NBRC 100779[T] (97.85%)	M	–	–	–	–	–	–	–	–	–	–	–	–
		A	1.91	–	–	–	–	–	–	–	–	–	–	–
7BTP-10	*Streptomyces qinglanensis*	E	–	–	–	–	0.64	0.57	0.69	–	–	–	–	–
(MG563312)	172205[T] (98.86%)	M	–	–	–	–	–	1.45	–	–	–	–	–	–
		A	–	–	–	–	–	–	–	–	0.64	-	–	–
10BTP-2	*Streptomyces scopuliridis*	E	–	–	–	–	0.54	-	0.54	0.60	–	–	0.93	0.80
(MG563314)	NRRL B-24574[T] (99.29%)	M	–	–	–	–	–	–	–	–	–	–	–	–
		A	2.23	0.56	0.69	–	–	1.61	–	–	–	–	–	–
6BTZ-4	*Streptomyces albogriseolus*	E	–	–	–	–	–	–	–	–	–	–	–	–
(MG563315)	NRRL B-1305[T] (99.86%)	M	–	–	–	–	–	–	–	–	–	–	–	–
		A	2.25	1.02	–	–	1.30	0.94	0.75	-	0.59	0.55	–	–
1BBP-1	*Streptomyces tunisiensis*	E	–	–	–	–	1.31	0.81	0.57	–	–	–	–	–

(MG563317)	CN-207^T (99.86%)	M	–	–	–	–	0.75	0.74	–	-	-	–	–	–
		A	–	–	–	–	-	–	–	–	-	–	–	–
7BMP-1	*Streptomyces*	E	1.40	-	–	–	1.91	1.61	1.38	1.60	–	–	0.97	-
(MG563368)	*phaeoluteichromatogenes*	M	–	–	–	–	1.77	1.77	-	1.27	–	–	–	–
	NRRL 5799^T (98.22%)	A	–	–	–	–	1.54	1.17	1.36	1.21	–	–	–	–
10BMP-1	*Streptomyces*	E	–	–	–	–	0.81	-	0.93	0.86	0.62	–	–	–
(MG563316)	*sundarbansensis* MS1/7^T	M	–	–	–	–	–	–	–	–	–	–	–	–
	(99.71%)	A	–	–	–	–	–	-	-	1.11	–	–	-	–
1BQY-1	*Streptomyces olivaceus*	E	–	–	–	–	0.86	-	-	–	–	–	–	–
(MG563318)	NRRL B-3009^T (100.00%)	M	–	–	–	–	–	–	–	–	–	–	–	–
		A	–	–	–	–	–	–	–	–	–	–	–	–
3BXP-1	*Streptomyces*	E	–	–	–	–	–	–	–	–	–	–	-	–
(MG563321)	*sundarbansensis*	M	–	–	–	–	–	–	–	–	–	–	–	–
	MS1/7^T (99.42%)	A	2.51	0.79	0.73	0.63	–	–	–	–	-	–	–	–
1BXP-1	*Streptomyces scopuliridis*	E	-	-	-	-	-	-	-	-	-	-	-	-
(MG563323)	NRRL B-24574^T (99.14%)	M	-	-	-	-	-	-	-	-	-	-	-	-
		A	1.98	0.90	-	-	-	-	-	-	1.05	-	-	
5BXP-6	*Streptomyces scopuliridis*	E	–	–	–	–	–	–	–	–	–	–	–	–
(MG563319)	NRRL B-24574^T (99.14%)	M	–	–	–	–	0.70	–	–	–	–	–	–	–
		A	2.09	-	-	-	-	–	–	–	-	–	–	–
8BXP-3	*Streptomyces puniceus*	E	–	–	–	–	1.93	1.68	2.01	-	–	–	0.96	0.81
(MG563320)	NRRL ISP-5058^T (99.57%)	M	–	–	–	–	0.95	0.84	–	-	–	–	–	–
		A	–	–	–	–	–	-	–	–	–	–	–	–
3BQP-1	*Streptomyces puniceus*	E	–	–	–	–	0.81	1.03	0.85	1.01	0.76	–	–	–
(MG563322)	NRRL ISP-5058^T (99.14%)	M	–	–	–	–	-	-	–	-	–	–	–	–
		A	–	–	–	–	–	–	-	–	–	–	–	–

1BMY-J3	*Curtobacterium*	E	–	–	–	–	–	–	–	–	–	–	–	–
(MG563331)	*flaccumfaciens* LMG 3645^T	M	–	–	–	–	–	–	–	–	–	–	–	–
	(99.86%)	A	–	–	-	-	–	–	–	–	-	–	–	–
9BXZ-J1	*Curtobacterium luteum*	E	–	–	–	–	–	–	–	–	–	–	–	–
(MG563330)	DSM 20542^T (99.57%)	M	–	–	–	–	–	–	–	–	–	–	–	–
		A	–	–	–	–	–	–	–	–	-	–	–	–
7BQZ-J1	*Curtobacterium luteum*	E	–	–	–	–	–	–	–	–	–	–	-	–
(MG563324)	DSM 20542^T (99.86%)	M	–	–	–	–	–	–	–	–	–	–	–	–
		A	–	–	–	–	–	-	–	–	–	–	–	–
4BXG-J1	*Curtobacterium albidum*	E	–	–	–	–	–	–	-	–	–	–	-	–
(MG563327)	DSM 20512^T (98.86%)	M	–	–	–	–	–	–	–	–	–	–	–	–
		A	–	–	–	–	–	–	–	–	–	–	–	–
1BMY-J1	*Curtobacterium luteum*	E	–	–	–	–	–	–	–	–	–	–	1.07	0.69
(MG563325)	DSM 20542^T (99.71%)	M	–	–	–	–	–	–	–	–	–	–	–	–
		A	–	–	–	–	–	–	–	–	–	–	–	–
6BMY-J2	*Curtobacterium*	E	–	–	–	–	–	–	–	–	–	–	-	–
(MG563326)	*flaccumfaciens* LMG 3645^T	M	–	–	–	–	–	–	–	–	–	–	–	–
	(99.86%)	A	–	–	-	–	–	–	–	–	–	–	–	–
10BMY-1	*Curtobacterium pusillum*	E	–	–	–	–	–	–	–	–	–	–	–	–
(MG563328)	DSM 20527^T (99.86%)	M	–	–	–	–	–	–	–	–	–	–	–	–
		A	–	–	–	–	–	–	–	–	–	–	–	-
7BXG-2	*Curtobacterium citreum*	E	–	–	–	–	–	–	–	–	–	–	1.00	0.72
(MG563333)	DSM 20528^T (99.57%)	M	–	–	–	–	–	–	–	–	–	–	–	–
		A	1.28	—	—	—	—	–	–	—	–	–	–	–
10BXG-1	*Curtobacterium citreum*	E	–	–	–	–	–	–	–	–	–	–	–	–
(MG563329)	DSM 20528 ^{T} (99.71%)	M	–	–	–	–	–	–	–	–	–	–	–	–

		A	–	–	–	–	–	–	–	–	–	–	–	–
9BXG-3	*Curtobacterium pusillum*	E	–	–	–	–	–	–	–	–	–	–	–	–
(MG563332)	DSM 20527 ^{T} (99.71%)	M	–	–	–	–	–	–	–	–	–	–	–	–
		A	–	–	–	–	–	–	–	–	–	–	–	–
8BTY-J2	*Micrococcus aloeverae*	E	–	–	0.70	-	1.11	0.76	0.93	-	–	–	1.38	0.84
(MG563335)	AE-6 ^{T} (99.71%)	M	–	–	–	–	–	–	–	–	–	–	–	–
		A	0.86	-	–	–	–	–	–	–	-	–	–	–
9BBP-J1	*Micrococcus flavus*	E	–	–	–	–	–	–	–	–	–	–	–	–
(MG563334)	LW4 ^{T} (99.29%)	M	–	–	–	–	–	–	–	–	–	–	–	–
		A	–	–	–	–	–	–	–	–	–	–	–	–
5BBZ-1	*Micrococcus endophyticus*	E	–	–	–	–	–	–	–	–	–	–	-	–
(MG563336)	YIM 56238 ^{T} (99.86%)	M	–	–	–	–	–	–	–	–	–	–	–	–
		A	–	–	–	–	–	–	–	–	–	–	–	–
10BTP-3	*Nocardioides aquiterrae*	E	–	–	–	–	–	–	–	–	–	–	–	–
(MG563370)	GW-9^T (97.44%)	M	–	–	–	–	–	–	–	–	–	–	–	–
		A	–	–	–	–	–	–	–	–	–	–	–	–
6BMS-2	*Nocardioides soli*	E	–	–	–	–	–	–	–	–	–	–	–	–
(MG563371)	mbc-2^T (96.99%)	M	–	–	–	–	–	–	–	–	–	–	–	–
		A	–	–	–	–	–	–	–	–	–	–	–	–
7BQP-J1	*Nocardioides ginkgobilobae*	E	–	–	–	–	–	–	–	–	–	–	–	–
(MG563337)	SYP-A7303^T (99.0%)	M	–	–	–	–	–	–	–	–	–	–	–	–
		A	1.97	-	–	–	–	–	–	–	–	–	–	–
9BQZ-J1	*Nocardioides sonneratiae*	E	–	–	—	–	–	–	–	–	–	–	–	–
(MG563366)	BGMRC0092^T (99.93%)	M	—	—	—	—	—	—	—	—	—	—	—	—
		A	–	–	–	–	–	–	–	–	–	–	–	–

1BMS-J1	*Mycobacterium poriferae*	E	–	–	–	–	–	–	–	–	–	–	–	–
(MG563338)	ATCC 35087^T (99.43%)	M	–	–	–	–	–	–	–	–	–	–	–	–
		A	1.80	–	0.58	–	–	–	–	-	–	–	–	–
3BMS-J1	*Mycobacterium peregrinum*	E	–	–	–	–	–	–	–	–	–	–	–	–
(MG563369)	ATCC 14467^T (98.55%)	M	–	–	–	–	–	–	–	–	–	–	–	–
		A	–	–	–	–	–	–	–	–	–	–	–	–
1BMY-J2	*Kocuria palustris*	E	–	–	–	–	–	–	–	–	–	–	–	–
(MG563339)	DSM 11925^T (100%)	M	–	–	–	–	–	–	–	–	–	–	–	–
		A	–	–	–	–	–	–	–	–	–	–	–	–
5BXY-2	*Kocuria rosea*	E	–	–	–	–	–	–	–	–	–	–	–	–
(MG563340)	DSM 20447^T (99.71%)	M	-	0.62	–	–	0.88	–	0.74	-	0.74	-	-	-
		A	–	–	–	–	–	–	–	–	–	–	–	–
1BMY-1	*Brevibacterium casei*	E	–	–	–	–	–	–	–	–	–	–	–	–
(MG563341)	NCDO 2048^T (100%)	M	–	–	–	–	–	–	–	–	–	–	–	–
		A	–	–	–	–	–	–	–	–	–	–	–	–
5BMY-2	*Brevibacterium celere*	E	–	–	–	–	–	–	–	–	–	–	–	–
(MG563342)	KMM 3637^T (99.71%)	M	–	–	–	–	–	–	–	–	–	–	–	–
		A	–	–	–	–	–	–	–	–	–	–	–	–
2BQY-1	*Brevibacterium casei*	E	–	–	–	–	–	–	–	–	–	–	–	–
(MG563343)	NCDO 2048^T (100%)	M	–	–	–	–	–	–	–	–	–	–	–	–
		A	–	–	–	–	–	–	–	–	–	–	–	–
4BXY-1	*Microbacterium maritypicum*	E	–	–	–	–	–	–	–	–	–	–	0.99	–
(MG563344)	DSM 12512^T (99.71%)	M	–	–	–	–	–	–	–	–	–	–	–	–
		A	–	–	–	–	-	–	–	–	–	–	–	–
5BTY-3	*Pseudokineococcus marinus*	E	–	–	–	–	–	–	–	–	–	–	–	–
(MG563345)	KST3-3^T (99.0%)	M	–	–	–	–	–	–	-	–	–	–	–	–

		A	–	–	–	–	–	–	–	–	–	–	–	–
5BTZ-J1	*Kineococcus endophytica*	E	–	–	–	–	–	–	–	–	–	–	–	–
(MG563346)	KLBMP 1274^{T} (98.98%)	M	–	–	–	–	–	–	–	–	–	–	–	–
		A	–	–	–	–	-	–	–	–	–	–	–	–
4BMP-J1	*Micromonospora chalcea*	E	–	–	–	–	1.32	1.02	–	–	–	–	–	–
(MG563347)	DSM 43026^{T} (99.71%)	M	–	–	–	–	–	–	–	–	–	–	–	–
		A	1.54	0.64	–	–	–	–	–	-	–	–	–	–
4BMS-1	*Micromonospora fluostatini*	E	–	–	–	–	0.86	0.85	1.38	0.68	0.68	0.74	-	–
(MG563348)	PWB-003^{T} (99.0%)	M	0.79	-	–	–	0.79	-	–	0.82	–	–	–	–
		A	1.70	-	0.58	0.62	-	-	–	–	–	–	–	–
6BXZ-1	*Agrococcus jejuensis*	E	–	–	–	–	–	–	–	–	–	–	–	–
(MG563349)	SSW1-48^{T} (99.14%)	M	–	–	–	–	–	–	–	–	–	–	–	–
		A	–	–	–	–	–	–	–	–	–	–	–	–
1BBY-J2	*Brachybacterium squillarum*	E	–	–	–	–	–	–	–	–	–	–	–	–
(MG563350)	M-6-3^{T} (99.0%)	M	–	–	–	–	–	–	–	–	–	–	–	–
		A	–	–	–	–	–	–	–	–	–	–	–	–
2BQY-J4	*Sanguibacter marinus*	E	–	–	–	–	–	–	–	–	–	–	–	–
(MG563351)	1-19^{T} (100%)	M	–	–	–	–	–	–	–	–	–	–	–	–
		A	–	–	–	–	–	–	–	–	–	–	–	–
5BXY-3	*Isoptericola nanjingensis*	E	–	–	–	–	–	–	–	–	–	–	–	–
(MG563352)	H17^{T} (99.57%)	M	–	–	–	–	–	–	–	–	–	–	–	–
		A	–	–	–	–	–	–	–	–	–	–	–	–
1BXZ-J1	*Marmoricola aequoreus*	E	–	–	–	–	–	–	–	–	–	–	–	–
(MG563365)	NRRL B-24464^{T} (96.32 %)	M	–	–	–	–	–	–	–	–	–	–	–	–
		A	1.94	-	–	–	1.99		–	–	–	–	–	–

8BXZ-J1	*Marmoricola solisilvae*	E	–	–	–	–	-		–	0.80	–	–	-	–
(KY921599)	KIS18-7^T (96.93%)	M	–	–	–	–	–	–	–	–	–	–	–	–
		A	1.71	-	–	–	–	–	–	–	–	–	–	–
10BMS-J1	*Actinoplanes brasiliensis*	E	–	–	–	–	–	–	–	–	–	–	–	–
(MG563353)	DSM 43805^T (98.43%)	M	–	–	–	–	–	–	–	–	–	–	–	–
		A	–	–	–	–	–	–	–	–	–	–	–	–
1BXZ-1	*Leucobacter tardus*	E	–	–	–	–	–	–	–	–	–	–	–	–
(MG563354)	K70/01^T (99.86%)	M	–	–	–	–	–	–	–	–	–	–	–	–
		A	2.19	-	–	–	–	–	–	–	–	–	–	–
8BMY-J2	*Gordonia didemni*	E	–	–	–	–	–	–	–	–	–	–	–	–
(MG563355)	B204^T (98.55%)	M	–	–	–	–	–	–	–	–	–	–	–	–
		A	1.13	-	–	–	–	–	–	–	–	–	–	–
5BTG-J1	*Citricoccus nitrophenolicus*	E	–	–	–	–	–	–	–	–	–	–	–	–
(MG563356)	PNP1^T (99.86%)	M	–	–	–	–	–	–	–	–	–	–	–	–
		A	1.88	0.55	–	–	–	–	–	-	-	–	–	–
3BMZ-J1	*Amnibacterium kyonggiense*	E	–	–	–	–	–	–	–	–	–	–	–	–
(MG563372)	KSL51201-037^T (98.12%)	M	–	–	–	–	–	–	–	–	–	–	–	–
		A	–	–	–	–	–	–	–	–	–	–	–	–
2BXY-2	*Kytococcus schroeteri*	E	–	–	–	–	–	–	–	–	–	–	–	–
(MG563357)	DSM 13884^T (99.71%)	M	–	–	–	–	–	–	–	–	–	–	–	–
		A	2.17	-	–	–	1.46	0.79	-	-	–	–	–	–
2BXP-3	*Kytococcus sedentarius*	E	–	–	–	–	–	–	–	–	–	–	–	–
(MG563358)	DSM 20547^T (99.86%)	M	–	–	–	–	–	–	–	–	–	–	–	–
		A	–	–	–	–	–	–	–	–	–	–	–	–
1BXP-5	*Dermacoccus profundi*	E	–	–	–	–	–	–	–	–	–	–	-	-

(MG563359)	MT2.2^T (100%)	M	–	–	–	–	–	–	–	–	–	–	–	–
		A	–	–	–	–	–	–	–	-	–	–	–	–
2BMS-1	*Nocardia araoensis*	E	–	–	–	–	–	–	–	–	–	–	–	–
(MG563360)	NBRC 100135^T (99.57%)	M	–	–	–	–	–	–	–	–	–	–	–	–
		A	2.33	0.62	0.73	-	–	–	–	-	0.55	–	–	–
6BMS-4	*Verrucosispora gifhornensis*	E	–	–	–	–	–	-	–	–	–	–	–	–
(MG563361)	DSM 44337^T (100%)	M	–	–	–	–	–	–	–	–	–	–	–	–
		A	–	–	–	–	–	–	–	–	–	–	–	–
5BXG-4	*Glutamicibacter nicotianae*	E	–	–	–	–	–	–	–	–	–	–	–	–
(MG563362)	DSM 20123^T (100%)	M	–	–	–	–	–	–	–	–	–	–	–	–
		A	–	–	–	–	–	–	–	–	–	–	–	–
1BXP-3	*Janibacter melonis*	E	–	–	–	–	–	–	–	–	–	–	–	–
(MG563363)	CM2104^T (99.86%)	M	–	–	–	–	–	–	–	–	–	–	–	–
		A	–	–	–	–	–	–	–	–	–	–	–	–
2BMD-3	*Nocardiopsis dassonvillei*	E	–	–	–	–	–	–	–	–	–	–	–	–
(MG563364)	DSM 43111^T (99.71%)	M	–	–	–	–	–	–	–	–	–	–	–	–
		A	1.58	–	–	–	–	–	–	–	–	–	–	–
		methanol	–	–	–	–	–	–	–	–	–	–	–	–
		levofloxacin	1.18	0.87	1.25	1.10	1.07	0.76	1.57	0.93	2.02	1.18	2.73	2.09

[a]E: crude sample extracted with ethyl acetate; M: crude sample from mycelium; A: crude sample from water layer;

[b]The diameters of the inhibition zones: cm; −, no inhibition.
